# Production of schizophyllan from distiller’s dried grains with solubles by diverse strains of *Schizophyllum commune*

**DOI:** 10.1186/2193-1801-2-476

**Published:** 2013-09-22

**Authors:** Nongnuch Sutivisedsak, Timothy D Leathers, Neil PJ Price

**Affiliations:** U.S. Department of Agriculture, Renewable Product Technology Research Unit, National Center for Agricultural Utilization Research, Agricultural Research Service, 1815 North University Street, Peoria, IL 61604 USA

**Keywords:** Distiller’s dried grains with solubles, DDGS, *Schizophyllum commune*, Schizophyllan

## Abstract

Eleven diverse strains of *Schizophyllan commune* were examined for production of the biopolymer schizophyllan from agricultural biomass. Strains were grown in malt extract (ME) basal medium containing 1% (w/v) distiller’s dried grains with solubles (DDGS), an abundant coproduct of fuel ethanol production by the dry grind process. Ten of 11 strains tested produced more than 2 g schizophyllan/L. Two strains, ATCC 20165 and CBS 266.60, produced more than 10 g schizophyllan/L. Schizophyllan from these strains was similar to commercial product in terms of solution viscosity, molecular weight, and surface tension properties, suggesting that they would be equivalent in biomaterial applications.

## Background

**Figure 1:**
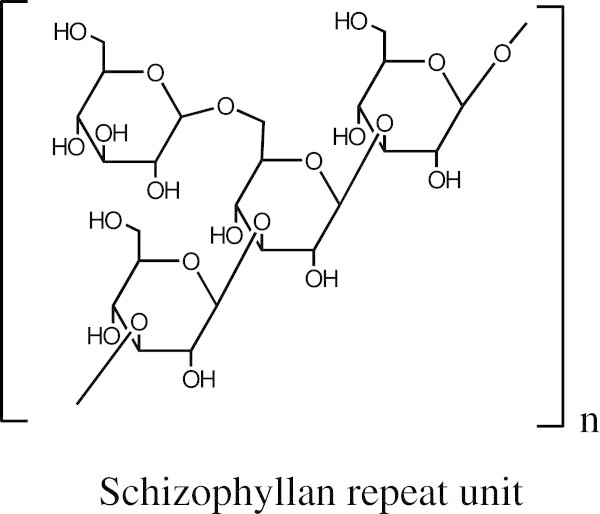
**Chemical structure of the schizophyllan repeat unit.**

Schizophyllan is a homoglucan with a β-1,3-linked backbone and single β-1,6-linked glucose side chains at every third residue (Figure 1), produced by the fungus *Schizophyllum commune* (Rau 1999; Rau 2002). Schizophyllan is commercially produced for use in anti-cancer therapies and as a bioactive cosmetics ingredient (Rau 2002). However, schizophyllan also has unique physical properties of high viscosity, film formation, and thermal stability that suggest bulk biomaterials applications. Schizophyllan can form oxygen-impermeable films for food preservation (Schulz et al. 1992), and it has been tested for use in enhanced petroleum recovery (Rau et al. 1992b).

Although glucose is used in conventional production of schizophyllan, *S. commune* can utilize a number of sugars and agricultural residues for polysaccharide production (Steiner et al. 1987; Leathers et al. 2006; Gao and Zhou 2008; Kumari et al. 2008; Shu and Hsu 2011). Inexpensive agricultural biomass residues could be appropriate for production of schizophyllan for biomaterials applications. Schizophyllan could fit into the integrated biorefinery concept as a value-added bioproduct from biomass. One readily available type of agricultural biomass is distiller’s dried grains with solubles (DDGS), an abundant coproduct of fuel ethanol production from corn by the dry-grind process (Rosentrater et al. 2012). We recently demonstrated the efficient utilization of DDGS for schizophyllan production by *S. commune* commercial production strain ATCC 38548 (Sutivisedsak et al. 2013).

Despite the fact that *S. commune* is an ubiquitous mushroom found world-wide, commercial production and most research studies have involved a single strain, ATCC 38548 (Prokop et al. 1992; Rau et al. 1992a; Sanroman and Nunez 1993; Shu and Hsu 2011). Few additional strains have been studied for schizophyllan production, such as NRCM and CGMCC 5.113 (Kumari et al. 2008; Li et al. 2011). In the current study we compare 11 diverse strains of *S. commune* for their ability to produce schizophyllan in medium containing DDGS.

## Results and discussion

### Production of schizophyllan by diverse strains of *Schizophyllum commune*

A set of 11 *S. commune* strains was chosen for this study, representing diverse isolation sources and sites (Table 1). Only non-clinical isolates were used because the goal was to identify strains with potential for industrial production. Ten of these strains produced more than 2 g schizophyllan/L in ME basal medium containing 1% (w/v) DDGS (Table 2). Thus, the capacity to produce schizophyllan from agricultural biomass appears to be a common trait among strains of *S. commune*. Two of these strains, ATCC 20165 and CBS 266.60, produced more than 10 g schizophyllan/L (Table 2). These yields are comparable to those obtained from commercial production strain ATCC 38548 (Sutivisedsak et al. 2013). Schizophyllan preparations from strains ATCC 20165 and CBS 266.60 were further examined for solution viscosity, molecular weight, and surface tension properties.

**Table 1:** **Strains of** ***Schizophyllum commune*** **used in this study**

Strain	Substrate of isolation	Depositors	Country	Designation	Synonym
ATCC 20165		Kyowa Ferm. Ind. Co., Ltd.		6 F2	
ATCC 26262		CA Raper		699	
ATCC 26890		Y Koltin		700	
ATCC 28095	Loblolly pine log	ER Toole		R-8	
ATCC 44200		J.G.H. Wessels, Jun 1981	USA	4-39	CBS 341.81
CBS 199.27	Conserved stem, Hevea	A. van Luijk, Koloniaal Inst., May 1927			MUCL 1008
CBS 249.69	In sawmill, Intsia bijuga	M.R. Monsalud, Univ. of the Philippines, Mar 1969	Philippines		Daedalea elegans, FPRI10
CBS 266.60	Mahogany wood	IMI, Mar 1960	UK		FPRL 9, IMI 061312
CBS 579.83	Decaying jute cutting	H. Esterbauer, Oct 1983			
CBS 109645	Litter	López, Aug 2001	Colombia		
NRRL A-23867		P.A. Lemke			
ATCC 38548	Elm twig	M.G. Paice		Delmar

**Table 2:** **Total biomass and production of schizophyllan (dry weights) by strains of** ***Schizophyllum commune*** ^**a**^

Strain	Total biomass (g/l)	Schizophyllan (g/l)
ATCC 20165	6.4 ± 0.5	10.1 ± 0.7
ATCC 26262	8.7 ± 0.1	8.3 ± 0.5
ATCC 26890	12.4 ± 0.4	5.9 ± 0.5
ATCC 28095	9.4 ± 0.5	2.2 ± 0.2
ATCC 44200	13.2 ± 0.2	0.6 ± 0.1
CBS 199.27	10.7 ± 0.5	2.4 ± 0.1
CBS 249.69	11.5 ± 0.4	3.4 ± 0.1
CBS 266.60	4.7 ± 0.8	12.7 ± 0.1
CBS 579.83	12.3 ± 0.1	2.1 ± 0.2
CBS 109645	13.2 ± 0.4	2.1 ± 0.1
NRRL A-23867	10 ± 0.1	2.3 ± 0.2

^a^ Cultures grown for 8 days on 1% (w/v) distiller’s dried grains with solubles (DDGS) in malt extract basal medium.

### Properties of schizophyllan solutions from *S. commune* strains ATCC 20165 and CBS 266.60

**Figure 2:**
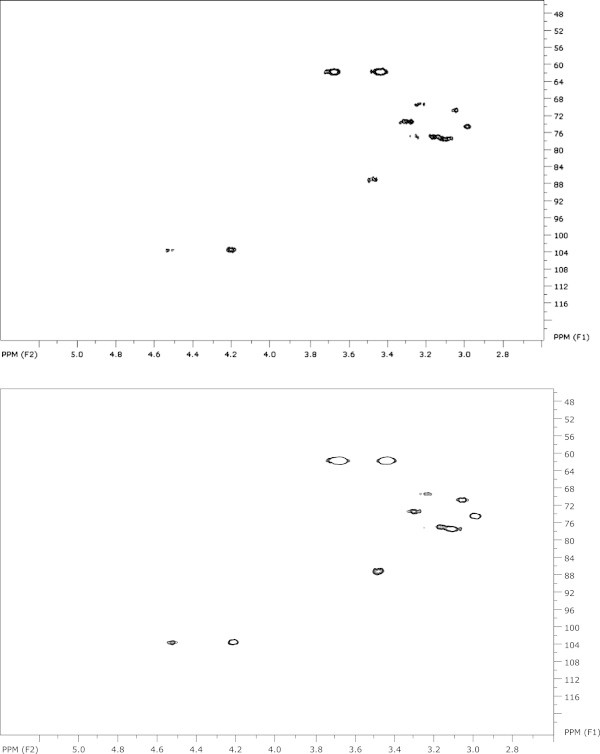
**HSQC 2D-NMR spectra of schizophyllans from strains CBS 266.6 (top) and ATCC 20165 (lower).**

Schizophyllan from *S. commune* strains ATCC 20165 and CBS 266.60 was structurally analyzed by Heteronuclear Single Quantum Coherence-NMR (Figure 2). The spectra were essentially identical to those of both a commercial schizophyllan standard and schizophyllan from commercial strain ATCC 38548. Spectra showed two anomeric sugar signals at 4.55 ppm and 4.20 ppm, due to the β-1,3-linked glucose and β-1,6-linked glucose, respectively. These signals correlated to overlapping ^13^C signals at 103.4 ppm, consistent with β-linked glucosyl residues. Other carbohydrate signals were apparent in the 2.7 – 4.0 ppm region for ^1^H, and 55 – 85 ppm for ^13^C nuclei. Characteristic methylene –CH_2_ signals were apparent at 3.40 and 3.60 ppm, coupled to a single ^13^C signal at 61 ppm. These were assigned to the C-6 position of the backbone glucose residues carrying a 1,6-glucose branch. These data are consistent with the isolated polysaccharides being schizophyllan.

**Figure 3:**
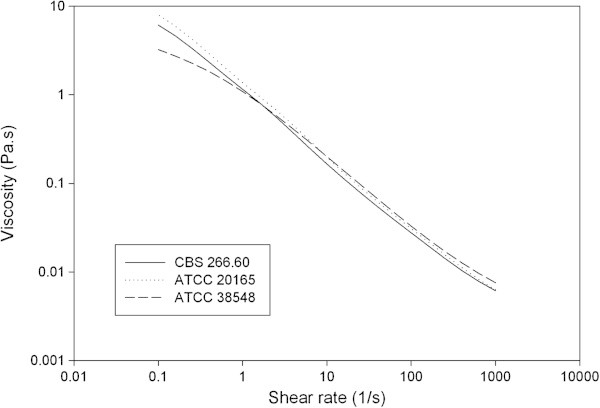
**Solution viscosity properties of 0.3% (w/v) aqueous solutions of schizophyllan produced by** ***Schizophyllum commune*** **on DDGS in ME basal medium.**

The solution viscosity properties of 0.3% (w/v) aqueous solutions of schizophyllan from strains ATCC 20165 and CBS 266.60 were compared with those of commercial strain ATCC 38548 (Figure 3). These strains exhibited nearly identical viscosity and shear thinning properties, characteristic of the pseudoplastic flow behavior of schizophyllan solutions (Rau 1999).

HPSEC was used to characterize the molecular weight of schizophyllan. Schizophyllan produced by *S. commune* strains ATCC 20165 and CBS 266.60 exhibited heterodisperse peaks with maximum molecular weights of 4.6 × 10^6^ and 5.2 × 10^6^, respectively. The values are consistent with literature values of 6–12 × 10^6^ (Rau 1999).

The interfacial tension of 0.3% (w/v) aqueous solutions of schizophyllan from *S. commune* strains ATCC 20165 and CBS 266.60 were 52 ± 1.9 and 55 ± 2.8 dy/cm, respectively (standard deviations shown). By comparison, schizophyllan from commercial strain ATCC 38548 showed similar values of 58 ± 3.5 dy/cm, while pure water exhibits an interfacial tension of 72 dy/cm (Dunlap et al. 2011). Thus, schizophyllan solutions from strains ATCC 20165 and CBS 266.60 showed relatively low surface activity, similar to commercial preparations (Rau 1999).

## Conclusions

Ten of 11 diverse strains of *S. commune* produced schizophyllan from agricultural biomass. Two of these strains, ATCC 20165 and CBS 266.60, produced yields comparable to those from a commercial production strain. The physical properties of schizophyllan solutions from these strains also were comparable to those of commercial schizophyllan, suggesting that they would be equivalent in biomaterial applications.

## Methods

### Strains and culture conditions

*Schizophyllum commune* strains used in this study were obtained from the American Type Culture Collection, Manassas, VA (ATCC strains), the ARS Culture Collection at the National Center for Agricultural Utilization Research, Peoria IL (NRRL strain) and the Centraalbureau voor Schimmelcultures, Utrecht, The Netherlands (CBS strains). Malt extract (ME) basal medium contained 2% (w/v) malt extract and 0.1% (w/v) peptone. Strains were grown on ME solid medium containing 2% (w/v) glucose and 2.5% (w/v) agar at 28°C for 7–10 days. A 7 mm × 7 mm square of mycelium was used to inoculate 250 mL of ME basal medium containing 2% (w/v) glucose in a 500 ml fluted Erlenmeyer flask with three 10 mm glass beads. This preinoculum culture was incubated at 240 rpm for 4–5 days at 30°C. Experimental cultures containing 1% (w/v) of DDGS in 150 mL of ME basal medium (without glucose) in 500 mL flasks were inoculated with 1.5 mL of preinoculum (1% v/v) and incubated at 240 rpm for 8 days at 30°C. DDGS was obtained from the National Corn-to-Ethanol Research Center, Edswardville, IL. All experiments were carried out in triplicate and standard deviations are shown.

### Isolation of schizophyllan

Whole culture suspensions were homogenized for 20 sec (Power Gen 700, Fisher Scientific) and centrifuged at 6,166 × g for 1 h at 4°C. The supernatants were collected and the insoluble pellets (containing mycelia and residual DDGS) were resuspended in 100 mL of deionized water, homogenized, and centrifuged as before. The pellets were dried under vacuum for 48 h at 60°C. The supernatants were combined and one volume of 95% ethanol was added. After 1 h at 4°C, precipitates were collected by centrifugation at 6,166 × g for 1 h at 4°C, air-dried overnight to reduce the amount of ethanol, and then lyophilized.

### NMR analysis

Solution NMR spectra were recorded on a Bruker AMX 500 spectrometer at normal probe temperature with standard instrument settings. Deuterated dimethylsulfoxide (d6-DMSO) was used as the solvent. All chemical shifts were referenced to tetramethylsilane at 0 ppm.

### Molecular weight determinations

Schizophyllan molecular weights were determined by size exclusion chromatography as previously described, using a Shodex SB-806 M high performance size exclusion chromatography (HPSEC) column (Showa Denko, Tokyo, Japan) and eluted with 0.05 M sodium nitrate at a flow rate of 0.5 ml/min (Leathers et al. 2010). The column was calibrated with a set of eight pullulan molecular weight standards ranging from 5.8 × 10^3^ Da to 1.66 × 10^6^ Da (Showa Denko, Tokyo, Japan). Separations were monitored using a Shodex OR-1 optical rotation detector (Showa Denko).

### Solution viscosity properties

Solution viscosity was measured using a TA Instruments (New Castle, DE) ARES LS-1 controlled strain rheometer with a 25 mm titanium parallel plate. All tests were performed at 25°C using a peltier plate. Steady rate sweeps were used to determine the viscosity of samples from 0.01-100 s^-1^. The Cross model was used to determine the zero shear viscosity, which was 460 Pa.s.

### Surface activity

Surface activity was determined using the pendant drop method (Dunlap et al. 2011). Samples were analyzed using the FTA 4000 surface tension instrument (First Ten Angstorms Inc., USA). Measurements were made using 22 gauge blunt needles with 7 μL drops. The reported values are the average of triplicate cultures.

## Abbreviations

DDGS: Distiller’s dried grains with solubles
d6-DMSO: Deuterated dimethylsulfoxide
HPSEC: High performance size exclusion chromatography
ME: Malt extract basal medium
NMR: Nuclear magnetic resonance
USDA: United States Department of Agriculture.
